# Union by Primary Intention

**Published:** 1902-07

**Authors:** Stuart McGuire

**Affiliations:** Richmond, Va.; Professor of Principles of Surgery and Clinical Surgery University College of Medicine; Surgeon in Charge St. Luke’s Hospital; Visiting Surgeon Virginia Hospital, etc.


### UNION BY PRIMARY INTENTION.*

BY STUART McGUIRE, M.D., RICHMOND, VA.

Professor of Principles of Surgery and Clinical Surgery University College of Medicine; Surgeon in Charge St. Luke's Hospital; Visiting Surgeon Virginia Hospital, etc.

All wounds heal by surgical regeneration, but union is said to occur by primary intention if suppuration is absent, and by secondary intention if pus is present.

*Read before the Richmond Academy of Medicine and Surgery, May 13, 1902.

Inflammation is undoubtedly due to germs, and text-books tell us that wounds that heal by primary intention are those which are aseptic, and those which heal by secondary intention are those which are infected.

This is obviously untrue, as experiments have proven that, as far as practical surgical work is concerned, absolute asepsis is an unattainable ideal. The bacteriologist has been able to demonstrate imperfections in the most rigid technique, and the result leads us to the conclusion that all wounds are infected.

If this be true, the manner in which a wound heals does not depend on the absence or presence of microbic life, but on the preponderance of the pathologic potency of the germ on the one hand, and the physiologic resistance of the tissues on the other. In other words, if the power of resistance is greater than the forces of attack inflammation does not develop and the wound heals by first intention; while if the force of attack is greater than the power of resistance inflammation does develop and the wound heals by secondary intention.

The question of how to secure union without suppuration is therefore reducible to a mathematical proposition: lessen the potency of the germ, or increase the resistance of the tissue; practically endeavor to do both. The potency of the germs must be estimated both qualitatively and quantitatively. It is to be minimized by the methods of modern aseptic and antiseptic surgery. Fortunately the most virulent species of micro-organisms inhabit material that can be completely disinfected, and the germs that exist on soil not capable of absolute sterilization can be so reduced numerically as to be comparatively harmless.

It is not the object of this paper, however, to discuss the methods employed to destroy the life of germs, but the purpose is to call attention to the equally important subject of the means to preserve the resistance of the tissues. The writer believes that in surgery, at least, cleanliness comes before godliness; but he does not think that cleanliness is everything. So much has been said and written about infection and so little about resistance, that the surgeon, in his efforts to secure the one, frequently fails to endeavor to maintain the other. For the best results both objects should be constantly held in view, and one not sacrificed to the other without good and sufficient cause.

When infection takes place, the process that follows is commonly spoken of as a battle between cells; really it is more. The germs effecting localization undergo multiplication and produce toxins. The tissue meets the attack of the invader with phagocytes, alexins and antitoxins.

*Phagocytes* are nominally present in the blood. When suppuration threatens, their number is largely increased, as if nature by phagocytosis called for conscripts to defend her soil. Phagocytes grapple in physical hand-to-hand combat with the microbes. If the phagocyte is victor it kills the germ, takes it into its substance and utilizes it as food. If the germ is the conqueror it kills the phagocyte, enters its body and employs it as a culture medium.

*Alexins* are normally present in the blood serum. They are the most powerful of all the non-toxic germicides. They give the serum an antiseptic power, equivalent bulk for bulk with a 1-10,000 solution of bichloride of mercury. Alexins act on the microbes chemically, and perhaps are as potent in the inhibition or destruction of bacteria as are the phagocytes themselves.

*Antitoxins* are not found normally in the body, but are produced by the organism in some unknown way after infection has taken place. They have no effect on the germ, but on the toxin produced by the germ. Antitoxin combines with a toxin as an alkali does with an acid, neutralizing it and rendering it inert.

The constitutional effect of an infection depends on the proportion of the toxin formed by the microbe and the antitoxin formed by the system. If the antitoxin is deficient there is fever; if it is equivalent there is cure; if it is excessive there is immunity.

The importance of the physiologic resistance of tissue to infection is generally known, but its augmentation or even its preservation is frequently neglected by the surgeon, who, in his enthusiasm over the details of aseptic and antiseptic technique, treats the body as if it were a test-tube, unmindful of the fact that the fate of the wound depends not only on what he puts into it, but also on what he takes out of it. The highest degree of physiologic resistance of tissue is to be secured by proper constitutional treatment of the patient before the operation and by skillful manipulation and judicious management of the wound during the operation.

When time permits, a case should receive more preparation than the usual routine purgation, starvation and sterilization. The body should be acclimated to bed life by confinement; the nervous system should be fortified by moral, and if necessary, medicinal measures; the heart and lungs should be examined and faulty action or diseased conditions corrected; the digestive functions should receive careful attention and be put in good condition by diet or drugs; the emunctories, especially the kidneys, bowels and skin, should be investigated and if healthy should be made active; the blood should be demonstrated to contain its proper percentage of hemoglobin and correct proportion of corpuscular elements; freedom from constitutional diseases like malaria should be assured, and in fact the entire system raised to its highest possible perfection, for the physiologic resistance of the individual serums and cells depends on the functional activity and physical condition of the body as a whole.

The local treatment, or the manner of making and the method of managing a wound, is even more important than the constitutional treatment or the preparation of the patient. It is here that the personal element that goes to make a good surgeon comes into play, for a skilful operator with a poor technique will often get better results than a clumsy operator with a good technique.

Aseptic and antiseptic methods are the result of laboratory experiments and capable of definite expression. They are mechanical and can be mastered by any and practiced by all. Surgical skill, however, is different. It cannot be taught but must be acquired. The essential qualifications are rapidity, a trait gotten by heredity; manipulative dexterity, and art gained by practice; and surgical judgment, a talent developed by experience. While no fixed rules can be laid down, the following general statements are true:

A wound should be made quickly and closed quickly, as long exposure devitalizes the tissues and lessens their resistance. A wound should be sufficiently liberal to permit necessary manipulations through it without mechanical injury to its surface, as a long clean wound heals quicker than a short bruised one. A wound should have its hemorrhage arrested by the gentlest means that will prove effective. Fine sutures for the large vessels, torsion for the medium, and gauze pressure for the small ones. Bites of ligatures and bites of forceps are to be avoided when possible. A wound should not be irrigated unless contaminated, as the cells absorb fluid, become bibulous and edematous, and lose their vigor. A wound should not be disinfected unless certainly infected, as germicides kill cells as well as bacteria. If infected it should be left open and drained until inflammation ceases. A wound should be united so as to leave no dead spaces for the accumulation of fluid. Sutures should effect approximation without strangulation. A tight stitch kills, and is a more frequent cause of suppuration than lack of aseptic precaution.

---

### ROACH EXTERMINATOR.

The following have been recommended by various authorities: Professor C. V. Riley, entomologist of the Agricultural Department, says nothing is better than the powdered flowers of the various pyrethra, especially recommending one growing wild in California. Professor Riley also recommends borax in powder, either alone or with pyrethrum. Professor A. A. Hopkins, of the *Scientific American* staff, recommends the following:

| | |
|---|---|
| Borax, powdered | 90 parts. |
| Corn starch | 9 parts. |
| Red coloring matter | 1 part. |

A German authority recommends the following:

| | |
|---|---|
| Borax | 50 parts. |
| Pyrethrum flowers | 25 parts. |
| Sulphur | 12 parts. |
| Crude arsenic | 1 part. |
| Corn starch | 12 parts. |

Reduce to a powder and mix. Strew in the haunts of the roaches. Care must be taken in strewing the powder in pantries and places where articles of food are kept.—*National Druggist.*

